# Sequence spaces $M(\phi)$ and $N(\phi)$ with application in clustering

**DOI:** 10.1186/s13660-017-1333-z

**Published:** 2017-03-21

**Authors:** Mohd Shoaib Khan, Badriah AS Alamri, M Mursaleen, QM Danish Lohani

**Affiliations:** 1 Department of Mathematics, South Asian University, New Delhi, India; 2 Operator Theory and Applications Research Group, Department of Mathematics, Faculty of Science, King Abdulaziz University, P.O. Box 80203, Jeddah, 21589 Saudi Arabia; 3 Department of Mathematics, Aligarh Muslim University, Aligarh, 202002 India

**Keywords:** 40H05, 46A45, clustering, double sequence, k-means clustering, two-moon dataset

## Abstract

Distance measures play a central role in evolving the clustering technique. Due to the rich mathematical background and natural implementation of $l_{p}$ distance measures, researchers were motivated to use them in almost every clustering process. Beside $l_{p}$ distance measures, there exist several distance measures. Sargent introduced a special type of distance measures $m(\phi)$ and $n(\phi)$ which is closely related to $l_{p}$. In this paper, we generalized the Sargent sequence spaces through introduction of $M(\phi)$ and $N(\phi)$ sequence spaces. Moreover, it is shown that both spaces are *BK*-spaces, and one is a dual of another. Further, we have clustered the two-moon dataset by using an induced $M(\phi)$-distance measure (induced by the Sargent sequence space $M(\phi)$) in the k-means clustering algorithm. The clustering result established the efficacy of replacing the Euclidean distance measure by the $M(\phi)$-distance measure in the k-means algorithm.

## Introduction

Clustering is a well-known procedure to deal with an unsupervised learning problem appearing in pattern recognition. Clustering is a process of organizing data into groups called clusters so that objects in the same cluster are similar to one another, but are dissimilar to objects in other clusters [1]. The main contribution in the field of clustering analysis was the pioneering work of MacQueen [1] and Bezdek [2]. They had introduced highly significant clustering algorithms such as k-means [1] and fuzzy c-means [2]. Among all clustering algorithms, k-means is the simplest unsupervised clustering algorithm that makes use of a minimum distance from the center, and it has many applications in scientific and industrial research [3–6] (for more information about the k-means clustering algorithm, see Section 5). K-means algorithm is distance dependent, so its outputs vary with changing distance measures. Among all distance measures, a clustering process was usually carried out through the Euclidean distance measure [7], but many times it failed to offer good results. In this paper, we define $M(\phi)$- and $N(\phi)$-distance measure. Further, $M(\phi )$-distance is used to cluster two-moon dataset. The output result is compared with the result of Euclidean distance measure to show the efficacy of $M(\phi)$-distance over the Euclidean distance measure. $M(\phi )$ and $N(\phi)$-distance measures are the generalization of $m(\phi)$- and $n(\phi)$-distance measures introduced by Sargent [8] and further studied by Mursaleen [9, 10] (to know more about $m(\phi )$ and $n(\phi)$, refer to [8–10]). The $M(\phi)$ and $N(\phi)$ spaces are closely related to $l_{p}$ distance measures. $l_{p}$ measures and its variance are mostly used to solve the problems evolving in the fields of Market prediction [11], Machine Learning [12], Pattern Recognition [13], Clustering [20] etc.

Throughout the paper, by *ω* we denote the set of all real or complex sequences. Moreover, by $l_{\infty}$, *c* and $c_{0}$ we denote the Banach spaces of bounded, convergent and null sequences, respectively; and let $l_{p}$ be the Banach space of absolutely *p*-summable sequences with *p*-norm ${\Vert \cdot \Vert } _{p}$. For the following notions, we refer to [14, 15]. A double sequence $x = (x_{jk})$ of real or complex numbers is said to be bounded if $\Vert x \Vert _{\infty} < \infty$, the space of all bounded double sequences is denoted by $\mathcal{L}_{\infty}$. A double sequence $x = (x_{jk})$ is said to converge to the limit *L* in Pringsheim’s sense (shortly, convergent to *L*) if for every $\varepsilon> 0$, there exists an integer *N* such that $\vert x_{jk} - L\vert < \varepsilon$ whenever $j,k > N$. In this case *L* is called the *p*-limit of *x*. If in addition $x \in\mathcal{L}_{\infty}$, then *x* is said to be boundedly convergent to *L* in Pringsheim’s sense (shortly, *bp*-convergent to *L*). A double sequence $x = (x_{jk})$ is said to converge regularly to *L* (shortly, *r*-convergent to *L*) if *x* is *p*-convergent and the limits $x_{j}: = \lim_{k}x_{jk}$ ($j \in\mathbb {N} $) and $x^{k}: = \lim_{j}x_{jk}$ ($k \in\mathbb{N} $) exist. Note that in this case the limits $\lim_{j}\lim_{k}x_{jk}$ and $\lim_{k}\lim_{j}x_{jk}$ exist and are equal to the *p*-limit of *x*. In general, for any notion of convergence *ν*, the space of all *ν*-convergent double sequences will be denoted by $\mathcal{C}_{\nu}$ and the limit of a *ν*-convergent double sequence *x* by $\nu\textrm{-} \lim_{j,k}x_{jk}$, where $\nu\in\{ p,\mathit{bp},r\}$.

Let Ω denote a vector space of all double sequences with the vector space operations defined coordinate-wise. Vector subspaces of Ω are called double sequence spaces. Let us consider a double sequence $x = \{ x_{mn}\}$ and define the sequence $s = \{ s_{mn}\}$ via *x* by

$$s_{mn}: = \sum_{i,j}^{m,n} x_{ij}\quad (m,n \in\mathbb{N} ). $$

Then the pair $(x,s)$ and the sequence $s = \{ s_{mn}\}$ are called a double series and a sequence of partial sums of the double series, respectively. Let *λ* be the space of double sequences converging with respect to some linear convergence rule $\mu\textrm{-} \lim:\lambda\to \mathbb{R}$. The sum of a double series $\sum_{i,j = 1}^{\infty,\infty} x_{ij}$ with respect to this rule is defined by $\mu\textrm{-} \sum_{i,j = 1}^{\infty,\infty} x_{ij}: = \mu\textrm{-} \lim s_{mn}$. Başar and Şever introduced the space $L_{p}$ in [16]

$$L_{p}: = \biggl\{ \{ x_{mn}\} \in\Omega:\sum _{m,n} \vert x_{mn}\vert ^{p} < \infty \biggr\} \quad (1 \le p < \infty) $$

corresponding to the space $l_{p}$ for $p \ge1$ and examined some of its properties. Altay and Başar [17] have generalized the spaces of double sequences $L_{\infty}$, $C_{p}$ and $C_{\mathit{bp}}$ to

$$\begin{aligned}& L_{\infty} (t) = \Bigl\{ \{ x_{mn}\} \in\Omega:\sup _{m,n \in\mathbb{N}} \vert x_{mn}\vert ^{t_{mn}} < \infty \Bigr\} , \\& C_{p}(t) = \Bigl\{ \{ x_{mn}\} \in\Omega:p \textrm{-} \lim _{m,n \to \infty} \vert x_{mn} - \ell \vert ^{t_{mn}} = 0 \Bigr\} , \end{aligned}$$

and

$$C_{\mathit{bp}}(t) = C_{p}(t) \cap L_{\infty} (t), $$

respectively, where $t = \{ t_{mn}\}$ is the sequence of strictly positive reals $t_{mn}$. In the case $t_{mn} = 1$, for all $m,n \in\mathbb{N}$, $L_{\infty} (t)$, $C_{p}(t)$ and $C_{\mathit{bp}}(t)$ reduce to the sets $L_{\infty}$, $C_{p}$ and $C_{\mathit{bp}}$, respectively. Further, let *C* be the space whose elements are finite sets of distinct positive integers. Given any element *σ* of *C*, we denote by $c(\sigma)$ the sequence $\{ c_{n}(\sigma)\}$ which is such that $c_{n}(\sigma) = 1$ if $n \in \sigma$, $c_{n}(\sigma) = 0$ otherwise. Further, let

$$C_{s} = \Biggl\{ \sigma\in C:\sum_{n = 1}^{\infty} c_{n}(\sigma) \le s \Biggr\} $$

be the set of those *σ* whose support has cardinality at most *s*, and

$$\Phi= \biggl\{ \phi= \{ \phi_{n}\} \in\omega: \phi_{1} > 0,\Delta\phi_{n} \ge0\mbox{ and }\Delta \biggl( \frac{\phi_{n}}{n} \biggr) \le0\ (n = 1,2, \ldots) \biggr\} , $$

where $\Delta\phi_{n} = \phi_{n} - \phi_{n - 1}$ and $\phi _{0} = 0$.

For $\phi\in\Phi$, the following sequence spaces were introduced and studied in [8] by Sargent and further studied by Mursaleen in [9, 10]:

$$m(\phi) = \biggl\{ x = \{ x_{n}\} \in\omega:\sup _{s \ge 1} \sup_{\sigma\in C_{s}} \biggl( \frac{1}{\phi_{s}} \sum_{n \in\sigma} \vert x_{n}\vert \biggr) < \infty \biggr\} , $$

and

$$n(\phi) = \Biggl\{ x = \{ x_{n}\} \in\omega:\sup _{u \in S(x)} \Biggl( \sum_{m,n = 1,1}^{\infty,\infty} \vert u_{n}\vert \Delta\phi_{n} \Biggr) < \infty \Biggr\} . $$

### Remark 1.1

(i) The spaces $m(\phi)$ and $n(\phi)$ are *BK*-spaces with their usual norms.
(ii) If $\phi_{n} = 1$ ($n = 1,2,3,\ldots$), then $m(\phi) = l_{1}$ [$n(\phi) = l_{\infty} $], and if $\phi_{n} = n$ ($n = 1,2,3,\ldots$), then $m(\phi) = l_{\infty}$ [$n(\phi) = l_{1} $].
(iii) $l_{1} \subseteq m(\phi) \subseteq l_{\infty} $ [$l_{1} \subseteq n(\phi) \subseteq l_{\infty} $] for all $\phi\in\Phi$.
(iv) For any $\phi\in\Phi$, $m(\phi)\neq l_{p}$ [$n(\phi)\neq l_{q} $], $1 < p < \infty$.

In this paper, we define Sargent’s spaces for double sequences $x = \{ x_{mn}\}$. For this we first suppose *U* to be the set whose elements are finite sets of distinct elements of $\mathbb{N} \times\mathbb{N}$ obtained by $\sigma\times\varsigma$, where $\sigma\in C_{s}$ and $\varsigma\in C_{t}$ for each $s,t \ge1$. Therefore, any element *ζ* of *U* means $(m,n)$; $m \in\sigma$ and $n \in\varsigma$ having cardinality at most *st*, where *s* is the cardinality with respect to *m* and *t* is the cardinality with respect to *n*. Given any element *ζ* of *U*, we denote by $c(\zeta)$ the sequence $\{ c_{mn}(\zeta)\}$ such that

$$c_{mn}(\zeta) = \textstyle\begin{cases} 1; & \mbox{if } (m,n) \in\zeta,\\ 0; & \mbox{otherwise}. \end{cases} $$

Further, we write

$$U_{st} = \Biggl\{ \zeta\in U:\sum_{m,n = 1}^{\infty,\infty} c_{mn}(\zeta) \le st \Biggr\} $$

for the set of those *ζ* whose support has cardinality at most *st*; and

$$\Theta= \biggl\{ \phi= \{ \phi_{mn}\} \in\Omega: \phi_{11} > 0,\Delta_{11}\phi_{mn} \ge0\mbox{ and } \Delta_{11} \biggl( \frac{\phi_{mn}}{mn} \biggr) \le0\ (m,n = 1,2, \ldots) \biggr\} , $$

where $\Delta_{11}\phi_{mn} = \phi_{mn} - \phi_{m - 1,n} - \phi_{m,n - 1} + \phi_{m - 1,n - 1}$ and $\phi_{00}$, $\phi_{0m}$, $\phi_{n0} = 0$, $\forall m,n \in\mathbb{I}^{ +}$. Throughout the paper, we write $\sum_{m,n \in\zeta}$ for $\sum_{m \in \sigma} \sum_{n \in\varsigma}$, and $S(x)$ is used to denote the set of all double sequences that are rearrangements of $x = \{ x_{mn}\} \in \Omega$. For $\phi\in\Theta$, we define the following sequence spaces:

$$M(\phi) = \biggl\{ x = \{ x_{mn}\} \in\Omega: \Vert x \Vert _{M(\phi )} = \sup_{s,t \ge1}\sup_{\zeta\in U_{st}} \biggl( \frac{1}{\phi_{st}}\sum_{m,n \in\zeta} \vert x_{mn} \vert \biggr) < \infty \biggr\} $$

and

$$N(\phi) = \Biggl\{ x = \{ x_{mn}\} \in\Omega: \Vert x \Vert _{N(\phi)} = \sup_{u \in S(x)} \Biggl(\sum _{m,n = 1}^{\infty,\infty} \vert u_{mn}\vert \Delta_{11}\phi_{mn} \Biggr) < \infty \Biggr\} . $$

Then the distances between $x = \{ x_{mn}\}$ and $y = \{ y_{mn}\}$ induced by $M(\phi)$ and $N(\phi)$ can be expressed as

$$d_{M(\phi)} = \sup_{s,t \ge1}\sup_{\zeta \in U_{st}} \biggl( \frac{1}{\phi_{st}}\sum_{m,n \in\zeta} \vert x_{mn} - y_{mn}\vert \biggr) $$

and

$$d_{N(\phi)} = \sup_{u,v \in S(x)} \Biggl( \sum _{m,n = 1}^{\infty,\infty} \vert u_{mn} - v_{mn} \vert \Delta_{11}\phi_{mn} \Biggr). $$

### Remark 1.2

If $\phi_{st} = 1$ ($s,t = 1,2,3,\ldots$), then $M(\phi) = L_{1}$ [$N(\phi) = L_{\infty} $], and if $\phi_{st} = st$ ($s,t = 1,2,3,\ldots$), then $M(\phi) = L_{\infty}$ [$N(\phi) = L_{1}$].

We now state the following known results of [18] for single sequences (series) which can also be proved easily for double sequences (series).

### Lemma 1.1

*If the series* $\sum u_{n}x_{n}$ *is convergent for every* *x* *of a* *BK*-*space* *E*, *then the functional* $\sum_{n = 1}^{\infty} u_{n}x_{n}$ *is linear and continuous in E*.

### Lemma 1.2

*If* *E* *and* *F* *are* *BK*-*spaces*, *and if* $E \subseteq F$, *then there is a real number* *K* *such that*, *for all* *x* *of* *E*,

$$\Vert x \Vert _{F} \le K\Vert x \Vert _{E}. $$

## Properties of the spaces $M(\phi)$ and $N(\phi)$

### Theorem 2.1

*The space* $M(\phi)$ *is a* *BK*-*space with the norm*.

2.1 $$ \Vert x \Vert _{M(\phi)} = \sup_{s,t \ge 1}\sup _{\zeta\in U_{st}} \frac{1}{\phi_{st}} \biggl( \sum _{m,n \in\zeta} \vert x_{mn}\vert \biggr). $$

### Proof

It is a routine verification to show that $M(\phi)$ is a normed space with the given norm (2.1), and so we omit it. Now, we proceed to showing that $M(\phi)$ is complete. Let $\{ x^{l}\}$ be a Cauchy sequence in $M(\phi)$, where $x^{l} = \{ x_{mn}^{l}\}_{m,n = 1,1}^{\infty,\infty}$ for every fixed $l \in\mathbb{N}$. Then, for a given $\varepsilon> 0$, there exists a positive integer $n_{0}(\varepsilon) > 0$ such that

$$\bigl\Vert x^{l} - x^{r} \bigr\Vert _{M(\phi)} = \sup_{s,t \ge 1}\sup_{\zeta\in U_{st}}\frac{1}{\phi_{st}} \biggl( \sum_{m,n \in\zeta} \bigl\vert x_{mn}^{l} - x_{mn}^{r} \bigr\vert \biggr) < \varepsilon $$

for all $l,r > n_{0}(\varepsilon)$, which yields, for each fixed $s,t \ge 1$ and $\zeta\in U_{st}$,

2.2 $$ \sum_{m,n \in\zeta} \bigl\vert x_{mn}^{l} - x_{mn}^{r} \bigr\vert \le\varepsilon \phi_{11}\quad \mbox{for all }l,r > n_{0}(\varepsilon). $$

Therefore

2.3 $$ \biggl\vert \sum_{m,n \in\zeta} \bigl\vert x_{mn}^{l} \bigr\vert - \sum_{m,n \in\zeta} \bigl\vert x_{mn}^{r} \bigr\vert \biggr\vert < \varepsilon\phi_{11} \quad \mbox{for all }l,r > n_{ 0} ( \varepsilon). $$

This means that $\{ \sum_{m,n \in\zeta} \vert x_{mn}^{l}\vert \} _{l \in\mathbb{N}}$ is a Cauchy sequence in $\mathbb{R}$ for every fixed $s,t \ge1$ and $\zeta \in U_{st}$. Since $\mathbb{R}$ is complete, it converges, say

$$\sum_{m,n \in\zeta} \bigl\vert x_{mn}^{l} \bigr\vert \to\sum_{m,n \in\zeta} \vert x_{mn} \vert \quad \mbox{as }l \to\infty. $$

Since absolute convergence implies convergence in $\mathbb{R}$, hence

2.4 $$ \sum_{m,n \in\zeta} x_{mn}^{l} \to\sum _{m,n \in\zeta} x_{mn} \quad \mbox{as } l \to\infty. $$

Hence we have

2.5 $$ \lim_{l \to\infty} \biggl\Vert \sum_{m,n \in\zeta} x_{mn}^{l} - \sum_{m,n \in\zeta} x_{mn} \biggr\Vert _{M(\phi)} = 0. $$

Let $y^{l} = \sum_{m,n \in\zeta} \vert x_{mn}^{l}\vert $. Then $\{ y^{l}\} \in l_{\infty}$. Therefore

$$\sup_{l \in\mathbb{N}} \sum_{m,n \in\zeta} \bigl\vert x_{mn}^{l} \bigr\vert \le k. $$

Since $\sum_{m,n \in\zeta} \vert x_{mn}\vert \le\sum_{m,n \in \zeta} \vert x_{mn} - x_{mn}^{l}\vert + \sum_{m,n \in\zeta} \vert x_{mn}^{l}\vert \le\varepsilon \phi_{11} + k$, it follows that $x = \{ x_{mn}\} \in M(\phi)$. Since $\{ x^{l}\}_{l \in\mathbb{N}}$ was an arbitrary Cauchy sequence, the space $M(\phi)$ is complete. Now we prove that $M(\phi)$ has continuous coordinate projections $p_{mn}$, where $p_{mn}:\Omega\to K$ and $p_{mn}(x) = x_{mn}$. The coordinate projections $p_{mn}$ are continuous since $\vert x_{mn}\vert \le\sup_{s,t \ge1}\sup_{\zeta\in U_{st}}\phi_{st}\Vert x \Vert _{M(\phi)}$ for each $m,n \in \mathbb{N}$. □

### Remark 2.1

The space $N(\phi)$ is a *BK*-space with the norm

$$\Vert x \Vert _{N(\phi)} = \sup_{u \in S(x)} \Biggl( \sum _{m,n = 1}^{\infty,\infty} \vert u_{mn}\vert \Delta_{11}\phi_{mn} \Biggr). $$

### Lemma 2.1

(i) *If* $x \in M(\phi)$ [$x \in N(\phi) $] *and* $u \in S(x)$, *then* $u \in M(\phi)$ [$u \in N(\phi) $] *and* $\Vert u\Vert = \Vert x\Vert $.
(ii) *If* $x \in M(\phi)$ [$x \in N(\phi)$] *and* $\vert u_{mn}\vert \le \vert x_{mn}\vert $ *for every positive integer* *m*, *n*, *then* $u \in M(\phi)$ [$u \in N(\phi)$] *and* $\Vert u\Vert \le \Vert x\Vert $.

### Proof

(i) Let $x \in M(\phi)$, then $\sup_{s,t \ge1} \sup_{\zeta\in U_{st}}\frac{1}{\phi_{st}}\sum_{m,n \in\zeta} \vert x_{mn}\vert < \infty$. So, we have

$$\frac{1}{\phi_{st}}\sum_{m,n \in\zeta} \vert x_{mn} \vert < \infty \quad \mbox{for each }\zeta\in U_{st}\mbox{ and }s,t \ge1. $$

Since the sum of a finite number of terms remains the same for all the rearrangements,

$$\frac{1}{\phi_{st}}\sum_{m,n \in\zeta} \vert u_{mn} \vert = \frac{1}{\phi_{st}}\sum_{m,n \in\zeta} \vert x_{mn}\vert \quad \mbox{for each }u \in S(x)\mbox{ and }\zeta\in U_{st}, s,t \ge1. $$

Hence

$$\sup_{s,t \ge1}\sup_{\zeta\in U_{st}}\frac{1}{\phi_{st}}\sum _{m,n \in\zeta} \vert u_{mn}\vert = \sup _{s,t \ge1} \sup_{\zeta\in U_{st}}\frac{1}{\phi_{st}}\sum _{m,n \in\zeta} \vert x_{mn}\vert < \infty, $$

thus $u \in M(\phi)$ and $\Vert u\Vert = \Vert x\Vert $.

(ii) By using the definition, easy to prove. □

### Theorem 2.2

*For arbitrary* $\phi\in\Theta$, *we have* $\Delta_{11}\phi\in M(\phi)$ *and* $\Vert \Delta_{11}\phi \Vert _{M(\phi)} \le2$.

### Proof

Let *s* and *t* be arbitrary positive integers, let $\sigma,\varsigma\in U_{st}$, and let $\tau_{1}$, $\tau_{2}$ constitute the element of *σ* and *ς* exceed by *s* and *t* respectively, also from the definition we have $\Delta_{11}\phi\ge0$ and $\Delta_{11} ( \frac{\phi_{mn}}{mn} ) \le0$. Then

$$\begin{aligned} \sum_{n \in\sigma,m \in\varsigma} \vert \Delta_{11} \phi_{mn} \vert \le& \sum_{n = 1,m = 1}^{s,t} \Delta_{11}\phi_{mn} + \sum_{n \in \tau_{1},m \in\tau_{2}} \Delta_{11}\phi_{mn} \\ \leq& \phi_{st} + \sum_{n \in\tau_{1},m \in\tau_{2}} \biggl( \frac{\phi_{m - 1,n - 1}}{(m - 1)(n - 1)} \biggr) \\ \leq & \phi_{st} + \left . \textstyle\begin{array}{c@{\quad}c@{\quad}c@{\quad}c@{\quad}c} \frac{\phi_{st}}{st} & + \frac{\phi_{s,t + 1}}{s(t + 1)} & + \frac{\phi_{s,t + 2}}{s(t + 2)} & +. &. \\ \frac{\phi_{s + 1,t}}{(s + 1)t} & + \frac{\phi_{s + 1,t + 1}}{(s + 1)(t + 1)} & + \frac{\phi_{s + 1,t + 2}}{(s + 1)(t + 2)} & +. &. \\ + & + & + &. & \\ . &. &. &. & \end{array}\displaystyle \right \} \quad \max(st) \textrm{-terms} \\ \leq& \phi_{st} + st\frac{\phi_{st}}{st} = 2\phi_{st}. \end{aligned}$$

 □

### Lemma 2.2

*If* $x \in M(\phi)$ *and* $\{ c_{11},c_{12}, \ldots,c_{1n},c_{21},c_{22}, \ldots,c_{2n}, \ldots,c_{m1},c_{m2}, \ldots,c_{mn}\}$ *is a rearrangement of* $\{ b_{11},b_{12}, \ldots,b_{1n},b_{21},b_{22}, \ldots,b_{2n}, \ldots,b_{m1},b_{m2}, \ldots,b_{mn}\}$ *such that* $\vert c_{11}\vert \ge \vert c_{12}\vert \ge \cdots\ge \vert c_{1n}\vert $, $\vert c_{21}\vert \ge \vert c_{22}\vert \ge\cdots\ge \vert c_{2n}\vert ,\dots, \vert c_{m1}\vert \ge \vert c_{m2}\vert \ge\cdots\ge \vert c_{mn}\vert $ *and* $\vert c_{11}\vert \ge \vert c_{21}\vert \ge\cdots\ge \vert c_{m1}\vert $, $\vert c_{12}\vert \ge \vert c_{22}\vert \ge\cdots \ge \vert c_{m2}\vert ,\vert c_{n1}\vert \ge \vert c_{n2}\vert \ge\cdots\ge \vert c_{nm}\vert $, *then*

$$\sum_{i,j = 1,1}^{m,n} \vert b_{ij}x_{ij} \vert \le \Vert x\Vert _{M(\phi )}\sum_{i,j = 1,1}^{m,n} \vert c_{ij}\vert \Delta_{11}\phi_{ij}. $$

### Proof

In view of Lemma 2.1(i), it is sufficient to consider the case when $b_{ij} = c_{ij}$ ($i = 1,2, \ldots,m$; $j = 1,2, \ldots,n$). Then writing $X_{mn} = \sum_{i,j = 1}^{m,n} \vert x_{ij}\vert $, we get

$$\begin{aligned} \sum_{i,j = 1,1}^{m,n} \vert b_{ij}x_{ij} \vert =& \sum_{i = 1}^{m - 1} \sum _{j = 1}^{n - 1} \bigl( \vert c_{ij}\vert - \vert c_{i,j + 1}\vert - \vert c_{i + 1,j}\vert +\vert c_{i + 1,j + 1}\vert \bigr)X_{ij} +\vert c_{mn}\vert X_{mn} \\ \le& \Vert x\Vert _{M(\phi)}\sum_{i = 1}^{m - 1} \sum_{j = 1}^{n - 1} \bigl( \vert c_{ij}\vert - \vert c_{i,j + 1}\vert - \vert c_{i + 1,j}\vert + \vert c_{i + 1,j + 1}\vert \bigr) \phi_{ij} + \Vert x\Vert _{M(\phi)}\vert c_{mn} \vert X_{mn} \\ =& \Vert x\Vert _{M(\phi)}\sum_{i,j = 1,1}^{m,n} \vert c_{ij}\vert \Delta_{11}\phi_{ij}. \end{aligned}$$

Hence we have $\sum_{i,j = 1,1}^{m,n} \vert b_{ij}x_{ij}\vert \le \Vert x\Vert _{M(\phi)}\sum_{i,j = 1,1}^{m,n} \vert c_{ij}\vert \Delta_{11}\phi_{ij}$. □

### Theorem 2.3

*In order that* $\sum u_{ij}x_{ij}$ *be convergent* [*absolutely convergent*] *whenever* $x \in M(\phi)$, *it is necessary and sufficient that* $u \in N(\phi)$. *Further*, *if* $x \in M(\phi)$ *and* $u \in N(\phi)$, *then*

2.6 $$ \sum_{i,j = 1,1}^{\infty,\infty} \vert u_{ij}x_{ij} \vert \le \Vert u\Vert _{N(\phi)} \Vert x\Vert _{M(\phi)}. $$

### Proof

*Necessity*. We now suppose that $\sum u_{ij}x_{ij}$ is convergent whenever $x \in M(\phi)$, then from Lemma 1.1 we have

$$\Biggl\vert \sum_{i,j = 1,1}^{\infty,\infty} u_{ij}x_{ij} \Biggr\vert \le K \Vert x\Vert _{M(\phi)} $$

for some real number *K* and all *x* of $M(\phi)$. In view of Lemma 2.1(ii), we may replace $x_{ij}$ by $x_{ij} \operatorname{sgn} \{ u_{ij}\}$, obtaining

2.7 $$ \sum_{i,j = 1,1}^{\infty,\infty} \vert u_{ij}x_{ij} \vert \le K \Vert x\Vert _{M(\phi)}. $$

Let $v \in S(u)$. Then taking *x* to be a suitable rearrangement of $\Delta_{11}\phi$, it follows from Eq. (2.7) and Theorem 2.2 and Lemma 2.1(i) that

$$\sum_{i,j = 1,1}^{\infty,\infty} \vert v_{ij} \vert \Delta_{11}\phi_{ij} \le4K, $$

and thus $u \in N(\phi)$.

*Sufficiency*. If $x \in M(\phi)$ and $u \in N(\phi)$, it follows from Lemma 2.2 that for every positive integer *m* and *n*,

$$\sum_{i,j = 1,1}^{\infty,\infty} \vert u_{ij}x_{ij} \vert \le \Vert u\Vert _{N(\phi)} \Vert x\Vert _{M(\phi)}. $$

 □

### Theorem 2.4

*In order that* $\sum u_{mn}x_{mn}$ *be convergent* [*absolutely convergent*] *whenever* $x \in N(\phi)$, *it is necessary and sufficient that* $u \in M(\phi)$.

### Proof

Since sufficiency is included in Theorem 2.3, we only consider necessity. We therefore suppose that $\sum u_{mn}x_{mn}$ is convergent whenever $x \in N(\phi)$. By arguments similar to those used in Theorem 2.3, we may therefore have that

2.8 $$ \sum_{m,n = 1,1}^{\infty,\infty} \vert u_{mn}x_{mn} \vert \le K \Vert x\Vert _{N(\phi)} $$

for some real number *K* and all *x* of $N(\phi)$. Let $x = c(\zeta)$, where $\zeta\in U_{st}$. Then $x \in N(\phi)$, and

$$\Vert x\Vert _{N(\phi)} = \sup_{\xi\in U_{st}}\sum _{m,n \in\xi} \Delta_{11}\phi_{mn} \le4 \phi_{st}, $$

from Theorem 2.2 and Eq. (2.8) we have

$$\sum_{m,n \in\zeta} \vert u_{mn}\vert \le4K \phi_{st}\quad (\zeta\in U_{st}; s,t = 1,2,3, \ldots), $$

and thus $u \in M(\phi)$. □

## Inclusion relations for $M(\phi)$ and $N(\phi)$

### Lemma 3.1

*In order that* $M(\phi) \subseteq M(\psi)$ [$N(\phi) \supseteq N(\psi)$], *it is necessary and sufficient that*

$$\sup_{s,t \ge1} \biggl( \frac{\phi_{st}}{\psi_{st}} \biggr) < \infty. $$

### Proof

Since each of the spaces $M(\phi)$ and $N(\phi )$ is the dual of the other, by Theorems 2.3 and 2.4, the second version is equivalent to the first. Moreover, sufficiency follows from the definition of an $M(\phi)$ space. We therefore suppose that $M(\phi) \subseteq M(\psi)$. Since $\Delta\phi\in M(\phi)$, it follows that $\Delta \psi\in M(\psi)$, and hence we find that, for every positive integer $s,t \ge1$,

$$\phi_{st} = \sum_{i,j = 1,1}^{s,t} \Delta_{11}\phi_{ij} \le\psi_{st} \Vert \Delta \phi \Vert _{M(\psi)},\quad \mbox{where }\Delta= \Delta_{11}. $$

 □

### Theorem 3.1

(i) $L_{1} \subseteq M(\phi) \subseteq L_{\infty}$ [$L_{1} \subseteq N(\phi) \subseteq L_{\infty} $] *for all* *ϕ* *of* Θ.
(ii) $M(\phi) = L_{1}$ [$N(\phi) = L_{\infty} $] *if and only if* $\mathit{bp}\textit{-}\lim_{s,t}\phi_{st} < \infty$.
(iii) $M(\phi) = L_{\infty}$ [$N(\phi) = L_{1}$] *if and only if* $\mathit{bp}\textit{-}\lim_{s,t}(\phi_{st}/st) > 0$.

### Proof

We prove here the first version, while the second version follows by Theorems 2.3 and 2.4. Since $\phi_{11} \le\phi_{mn} \le mn\phi_{mn}$ for all *ϕ* of Θ, we have by Lemma 3.1 that (i) is satisfied. Further, from Lemma 3.1, it follows that $M(\phi) \subseteq L_{1}$ if and only if $\sup_{s,t \ge 1}\phi_{st} < \infty$, while $L_{\infty} \subseteq M(\phi)$ if and only if $\sup_{s,t \ge1}(\phi_{st}/st) < \infty$; since the sequences $\{ \phi_{st}\}$ and $\{ st/\phi_{st}\}$ are monotonic, (ii) and (iii) are also satisfied. □

### Theorem 3.2

*Suppose that* $1 < p < \infty$ *and* $\frac{1}{p} + \frac{1}{q} = 1$. *Then*

(i) *Given any* *ϕ* *of* Θ, $M(\phi)\neq L_{p}$ [$N(\phi )\neq L_{q}$].
(ii) *In order that* $L_{p} \subset M(\phi)$ [$N(\phi) \subset L_{q}$], *it is necessary and sufficient that* $\sup_{s,t \ge1} ( \frac{(st)^{1/q}}{\phi_{st}} ) < \infty$.
(iii) *In order that* $M(\phi) \subset L_{p}$ [$N(\phi) \supset L_{q}$], *it is necessary and sufficient that* $\Delta\phi\in L_{p}$.
(iv) $\bigcup_{\Delta\phi\in L_{p}} M(\phi) = L_{p}$ [$\bigcap_{\Delta\phi\in L_{p}} N(\phi) = L_{q} $].

### Proof

(i) Let us suppose that $M(\phi) = L_{p}$.

Then, by Lemma 1.2, there exist real numbers $r_{1}$ and $r_{2}$ ($r_{1} > 0$, $r_{2} > 0$) such that, for all *x* of $M(\phi)$,

$$r_{1} \Vert x\Vert _{L_{p}} \le \Vert x\Vert _{M(\phi)} \le r_{2} \Vert x\Vert _{L_{p}}. $$

Taking $x = c(\zeta)$, where $\zeta\in U_{st}$, we have that

$$r_{1}(st)^{\frac{1}{p}} \le\frac{st}{\phi_{st}} \le r_{2}(st)^{\frac{1}{p}}\quad (s,t = 1,2,3, \ldots), $$

and hence that

$$r_{1} \le\frac{(st)^{\frac{1}{q}}}{\phi_{st}} \le r_{2} \quad (s,t = 1,2,3, \ldots). $$

In view of Lemma 3.1, this implies that $M(\phi) = M(\psi)$, where $\psi= \{ (mn)^{\frac{1}{q}}\}$. Since $\Delta\psi\in M(\psi)$ by Theorem 2.2, but $\Delta\psi\notin L_{q}$, this leads to a contradiction. Hence (i) follows.

(ii) If $L_{q} \subset M(\phi)$, arguments similar to those used in the proof of (i) show that

3.1 $$ (st)^{1/q} \le K\phi_{st} \quad (s,t = 1,2,3, \ldots). $$

For sufficiency, we suppose that (3.1) is satisfied. Then, whenever $x \in L_{p}$ and $\zeta\in U_{st}$,

$$\sum_{m,n \in\zeta} \vert x_{mn}\vert \le \biggl( \sum_{m,n \in\zeta} \vert x_{mn}\vert ^{p} \biggr)^{\frac{1}{p}} \biggl( \sum_{m,n \in\zeta} 1 \biggr)^{\frac{1}{q}} \le \Vert x\Vert _{L_{q}}(st)^{\frac{1}{q}} < K\phi_{st}\Vert x\Vert _{L_{q}}, $$

and hence $x \in M(\phi)$. In view of (i), it follows that $L_{q} \subset M(\phi)$.

(iii) By Theorem 2.2, we have $\Delta\phi\in M(\phi)$. For sufficiency, we suppose that $\Delta\phi\in L_{p}$ and that $x \in M(\phi)$. Then $\{ u_{mn}\Delta_{11}\phi_{mn}\} \in L_{1}$ whenever $u \in L_{q}$, and it therefore follows from Lemma 2.2 that $\{ u_{mn}x_{mn}\} \in L_{1}$ whenever $u \in L_{q}$. Since $L_{p}$ is the dual of $L_{q}$ and since $M(\phi)\neq L_{p}$, it follows that $M(\phi) \subset L_{q}$.

(iv) By using (iii) we have $\bigcup_{\Delta\phi\in L_{p}} M_{\phi} \subseteq L_{p}$. Now, for obtaining the complementary relation $L_{p} \subseteq\bigcup_{\Delta\phi\in L_{p}} M_{\phi}$, let us suppose that $x \in L_{p}$. Then $\lim_{m,n \to\infty} x_{mn} = 0$, and hence there is an element *u* of $S(x)$ such that $\{ \vert u_{mn}\vert \}$ is a non-increasing sequence. If we take $\psi= \{ \sum_{i,j = 1,1}^{m,n} \vert u_{ij}\vert \}$, then it is easy to verify that $\psi\in \Theta$ and that $x \in M(\phi)$. Since $\Delta\psi\in L_{p}$, the complementary relation is satisfied. □

## Application of $M(\phi)$ and $N(\phi)$ in clustering

In this section, we implement a k-means clustering algorithm by using $M(\phi)$-distance measure. Further, we apply the k-means algorithm into clustering to cluster two-moon data. The clustering result obtained by the $M(\phi)$-distance measure is compared with the results derived by the existing Euclidean distance measures ($l_{2}$).

### Algorithm to compute $M(\phi)$ distance

Let $x = [x_{1},x_{2},x_{3}, \ldots,x_{n}]_{1 \times n}$ and $y = [y_{1},y_{2},y_{3}, \ldots,y_{n}]_{1 \times n}$ be two matrices of size $1 \times n$, and let $\phi_{m,n} = \phi_{1,n} = n$.

1. Calculate $a_{i} = \frac{1}{\phi_{1,i}}\vert x_{i} - y_{i}\vert $, $i = 1,2,3, \ldots,n$.
2. The $M(\phi)$-distance between *x* and *y* is *d*, where
  $$d = \max\{ a_{1},a_{1} + a_{2}, \ldots,a_{1} + a_{2} + \cdots+ a_{n}\}. $$

### K-means clustering algorithm for $M(\phi)$-distance measure

Let $X = [x_{1},x_{2},x_{3},\ldots,x_{n}]$ be the data set.

1. Randomly/judiciously select *k* cluster centers (in this paper we choose first *k* data points as the cluster center $y = [x_{1},x_{2},\ldots,x_{k}]$).
2. By using $M(\phi)$ or $N(\phi)$ distance measure (since both are dual of each other, in application point of view, we only consider $M(\phi)$), compute the distance between each data points and cluster centers.
3. Put data points into the cluster whose $M(\phi)$-distance with its center is minimum.
4. Define cluster centers for the new clusters evolved due to steps 1-3, the new cluster centers are computed as follows: $c_{i} = \frac{1}{k_{i}}\sum_{j = 1}^{k_{i}} x_{i}$, where $k_{i}$ denotes the number of points in the *i*th cluster.
5. Repeat the above process until the difference between two consecutive cluster centers reaches less than a small number *ε*.

### Two-moon dataset clustering by using $M(\phi)$-distance measure in k-means algorithm

**Figure 1:**
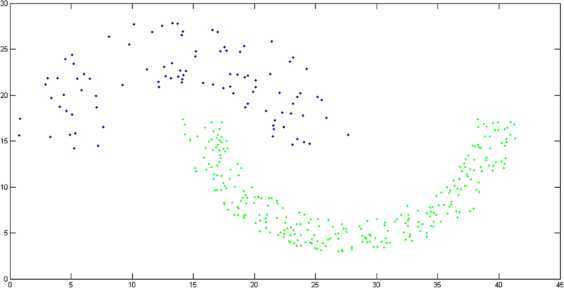
**Original shape of two-moon dataset.**

Two-moon dataset is a well-known nonconvex data set. It is an artificially designed two dimensional dataset consisting of 373 data points [19]. Two-moon dataset is visualized as moon-shaped clusters (see Figure 1).

**Figure 2:**
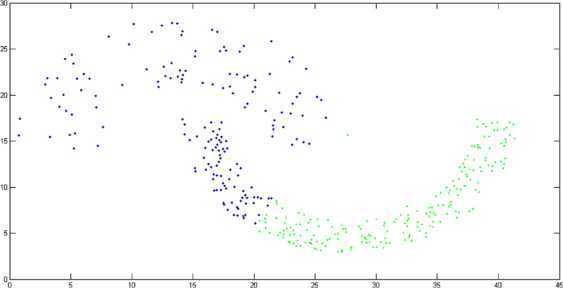
**Clustering induced by** $\pmb{M(\phi)}$ **-distance measure.**

**Figure 3:**
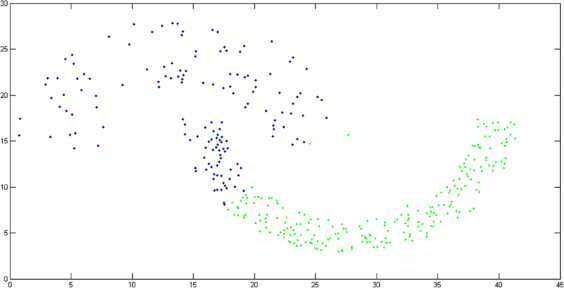
**Clustering induced by Euclidean distance** $\pmb{l_{2}}$ **.**

By using $M(\phi)$-distance measure in the k-means clustering algorithm, the obtained result is represented in Figure 2. In Figure 3, we represent the result obtained by using the Euclidean distance measure in the k-means algorithm (we measure the accuracy of the cluster by using the formula, accuracy = (number of data points in the right cluster/total number of data points)). The experimental result shows that cluster accuracy of $M(\phi)$-distance measure is 84.72% while $l_{2}$-distance measure’s clustering accuracy is 78.55%. Thus, $M(\phi)$-distance measure substantially improves the clustering accuracy.

## Conclusions

In this paper, we defined Banach spaces $M(\phi)$ and $N(\phi)$ with discussion of their mathematical properties. Further, we proved some of their inclusion relation. Furthermore, we applied the distance measure induced by the Banach space $M(\phi)$ into clustering to cluster the two-moon data by using the k-means clustering algorithm; the result of the experiment shows that the $M(\phi)$-distance measure extensively improves the clustering accuracy.
